# Supplementary material for: Investigating hill sheep farmers and crofters’ experiences of blackloss in the Highlands and Islands of Scotland
Source: PLoS One. 2024 Mar 27;19(3):e0298255. doi: 10.1371/journal.pone.0298255 (PMC10971779; doi:10.1371/journal.pone.0298255)
Supplement: S1 File — (PDF) [file pone.0298255.s001.pdf]

Investigating Causes of Lamb Loss on Highland Farms and Crofts

Study Information:
My name is Fiona McAuliffe and I am currently investigating causes of lamb loss on Highland farms and crofts as part of my PhD study with Scotland's Rural College and the University of Edinburgh. The information provided by hill sheep farmers and crofters in this questionnaire will form an important component of this PhD study. Blackloss is a term used for the unexplained losses of lambs on extensive hill grazings in the western highlands of Scotland. Working in collaboration with Scottish Natural Heritage the project will investigate possible causes of ill health and death of lambs, resulting in blackloss. By answering this questionnaire you will be contributing to identifying the causes of blackloss, which will assist in forming management strategies to reduce levels of blackloss on highland farms and crofts.

GDPR Statement:
All information provided will be protected under the terms of the General Data Protection Regulation (2018) which ensures that the data is securely recorded, used for a legitimate purpose and there is a compelling justification for processing the data. The survey is confidential. Your answers will be anonymised, and your personal details such as name and address will not be revealed to people outside of the project. Your individual answers will not be linked with your name or address in any reports of the data. Individuals will remain anonymous in any reports, presentations etc., and measures will be taken to avoid or minimise identification of participants if quotes are used. However, anonymity cannot be guaranteed, where illegal activity is mentioned and which may be reported to local authorities. Your participation is voluntary and if you come to any question you prefer not to answer, you are welcomed to skip it and go on to the next. If you have any questions regarding this questionnaire please contact me: Fiona.McAuliffe@sruc.ac.uk

Your help in gathering this important information is much appreciated.

Do you agree to take part in this survey? The data collected will be used to improve knowledge of causes of blackloss of lambs on Highland farms and crofts.

☐ I am happy to take part in this questionnaire.

☐ I am happy to be contacted about future developments in this project and to receive feedback about this questionnaire. This may involve being invited to participate in a more detailed study of lamb loss on your farm or croft.

Please note if you do not wish to take part in this questionnaire there is no requirement to return this form.

I, the undersigned, have read and understood the Study Information provided.
I have been given the opportunity to ask questions about the study.
I have been given adequate time to consider my decision and I agree to take part in the Study.
I understand that my personal details such as name and address will not be revealed to people outside of the project.
I understand that my words may be quoted in publications, reports, web pages, and other research outputs but my personal details will not be used.
For those providing contact details: I understand that until the data is anonymised I can withdraw from the Study and I will not be asked any questions about why I no longer want to take part.

Participant signature/initial: ________________________ Date: ___________
(Please initial if you wish to answer the questionnaire anonymously)

Thank you for taking the time to complete this survey.

Please return completed forms to:

Fiona Mc Auliffe,
SRUC, The Roslin Institute,
Easter Bush, Midlothian,
EH25 9RG

Or send a scanned copy to:

Fiona.McAuliffe@sruc.ac.uk

Section One: Your farm or croft

1. Type of system:

Farm	☐
Croft	☐
Other (*please specify*)	☐

2. Do you have access to common grazing? *(Circle one)*

Yes/No

3. Size of farm/croft *(Total hectares)*: ______ Ha

4. Which habitats are present on <u>your</u> farm/croft: (*please give estimates in hectares*)

Pasture grazing (Improved grassland/ In-bye)	
Rough grazing (semi-improved grassland)	
Mountain/hill (unimproved)	
Forestry/woodland	
Scrub	
Other (*please specify*)	

5. How much of the above is common grazing? ______ Ha

6. Do you have access to any of the following infrastructure/equipment on <u>your</u> farm/croft? *(Please select an answer for each)*

	Yes	Yes, shared	No
Livestock trailer	☐	☐	☐
Quarantine area	☐	☐	☐
Lambing shed	☐	☐	☐
Handling unit	☐	☐	☐
Race/Drafting pens	☐	☐	☐
Weigh scale	☐	☐	☐
Electronic ID tag stick reader	☐	☐	☐
EID stock recorder	☐	☐	☐
EID race/panel reader	☐	☐	☐
EID weigh crate	☐	☐	☐
EID auto drafter	☐	☐	☐
Other (*please specify*)	☐	☐	☐

7. Number of breeding ewes:

8. State the main ewe breeds on your farm/croft:

1	
2	
3	

9. Do you condition score your ewes: *(Circle one)*

Yes/No

→ **a. If *yes*, how many times a year do you condition score your ewes?** ______

10. Do you scan your ewes? *(Circle one)*

Yes/No

→ **a. If *yes*, what is your average scanning percentage:** ______%

11. How many weeks old are the majority of your lambs when they are marked/weaned?

Marking	weeks
Weaning	weeks

12. When do you tag the majority of your lambs *(select one)*?

- ☐ At lambing
- ☐ At marking
- ☐ At weaning
- ☐ When they leave the farm/are 9 months old
- ☐ Other (*please specify*)

13. For 2019, please report the number of lambs: *(If you are unsure of figures, please leave blank)*

At lambing	
At marking	
At weaning	
Tagged lambs	
Tags retrieved from dead lambs	
Lamb tags unaccounted for	
Lambs kept as replacements	
Lambs sold	

14. Where are the majority of your single and twin lambs grazed between marking and weaning?

	Singles	Twins
Pasture grazing (Improved grassland/ In-bye)	☐	☐
Rough grazing (semi-improved grassland)	☐	☐
Mountain/hill (unimproved)	☐	☐
Other (*please specify*)	☐	☐

Section Two: Blackloss on your farm or croft

15. In your opinion, what is 'blackloss'? *This question is very important in understanding what blackloss means to hill sheep farming and crofting communities. Please take your time in answering it.*

16. Do you therefore consider yourself to suffer from blackloss?

Yes/No

a. **If *yes*, please answer question 17, 18 and 19**
b. **If *no*, please skip directly to question 20**

17. Please give an estimate of the proportion of lambs you have lost to blackloss during the marking to weaning period in the past 3 years: ***(Select one for each year)***

	2017	2018	2019	Don't know
0%	☐	☐	☐	☐
1-10%	☐	☐	☐	☐
11-20%	☐	☐	☐	☐
21-30%	☐	☐	☐	☐
>30%	☐	☐	☐	☐

18. What do you think are the <u>most important causes</u> of blackloss of lambs on <u>your</u> farm/croft?
(For each line please indicate level of importance)

	Important	Slightly important	Not at all important	Don't know
Mismothering	☐	☐	☐	☐
Hypothermia/Exposure	☐	☐	☐	☐
Starvation	☐	☐	☐	☐
Predators	☐	☐	☐	☐
Parasites (worms, fluke, ticks)	☐	☐	☐	☐
Plochteach/Yellowses	☐	☐	☐	☐
Trace element deficiencies	☐	☐	☐	☐
Accidents	☐	☐	☐	☐
Theft	☐	☐	☐	☐
Other (*please specify*)	☐	☐	☐	☐

19. What do you think are the <u>main consequences</u> of blackloss on <u>your</u> farm/croft?
(Please select an answer for each in order of severity)

	Severe	Mild	None	Don't know
Loss of productivity	☐	☐	☐	☐
Farmer/crofter stress	☐	☐	☐	☐
Impact on animal welfare	☐	☐	☐	☐
Financial loss	☐	☐	☐	☐
Loss of breeding potential	☐	☐	☐	☐
Poor sustainability	☐	☐	☐	☐
Other (*please specify*)	☐	☐	☐	☐

<u>Section Three: Known causes of reduced lamb productivity up to weaning</u>

20. What percentage of your lambs suffer from the following diseases in an average year?
(Please select an answer for each)

	0%	1-20%	21-40%	41-60%	61-80%	81-100%	Don't know
Plochteach/Yellowses	☐	☐	☐	☐	☐	☐	☐
Trace element deficiencies	☐	☐	☐	☐	☐	☐	☐
Fluke	☐	☐	☐	☐	☐	☐	☐
Gastrointestinal worms	☐	☐	☐	☐	☐	☐	☐
Tick fever/ tick pyaemia/ louping ill	☐	☐	☐	☐	☐	☐	☐
Other (*please specify*)	☐	☐	☐	☐	☐	☐	☐

21. Have you recovered lamb carcasses on <u>your</u> farm/croft where the cause of death was determined?
Yes/No

→ **a. If *yes*, what was the most common cause of death?**
(Please rank 1=Most common cause of death, 2=Next most common… and so on)

	Mismothering
	Hypothermia/Exposure
	Starvation
	Trace element deficiencies (copper, cobalt, etc.)
	Parasites (worms, fluke, ticks)
	Plochteach/Yellowses
	Predators
	Accidents
	Other (*please specify*)

Section Four: Predator species on your farm or croft

22. What predator species are present on <u>your</u> farm/croft? *(Please select one answer for each species)*

	Yes	No	Don't know
Foxes	☐	☐	☐
Badgers	☐	☐	☐
Dogs	☐	☐	☐
Ravens	☐	☐	☐
Crows	☐	☐	☐
Black-backed gulls	☐	☐	☐
White tailed sea eagles	☐	☐	☐
Golden eagles	☐	☐	☐
Other (*Please specify*)	☐	☐	☐

23. Please select the level of impact those predators present on <u>your</u> farm/croft have on lambs which are <u>less than</u> 10 days old: *(Please select one answer for each predator)*

	High impact	Medium impact	Low impact	No impact	Not applicable
Foxes	☐	☐	☐	☐	☐
Badgers	☐	☐	☐	☐	☐
Ravens	☐	☐	☐	☐	☐
Crows	☐	☐	☐	☐	☐
Black-backed gulls	☐	☐	☐	☐	☐
White tailed sea eagles	☐	☐	☐	☐	☐
Golden eagles	☐	☐	☐	☐	☐
Other *(please specify)*	☐	☐	☐	☐	☐

24. Please select the level of impact those predators present on <u>your</u> farm/croft have on lambs which are <u>more than</u> 10 days old: *(Please select one answer for each predator)*

	High impact	Medium impact	Low impact	No impact	Not applicable
Foxes	☐	☐	☐	☐	☐
Badgers	☐	☐	☐	☐	☐
Ravens	☐	☐	☐	☐	☐
Crows	☐	☐	☐	☐	☐
Black-backed gulls	☐	☐	☐	☐	☐
White tailed sea eagles	☐	☐	☐	☐	☐
Golden eagles	☐	☐	☐	☐	☐
Other *(please specify)*	☐	☐	☐	☐	☐

25. Do you undertake predator control on <u>your</u> farm/croft? (*Circle one*)

Yes/No

→ **a. If *yes*, which predators do you control? (*Please tick all that apply*)**

- ☐ Fox control
- ☐ Crow control

26. In the past 3 years have you had problems with dogs worrying <u>your</u> sheep/lambs? (*Circle one*)

Yes/No

→ **a. If *yes*, how many were affected:** Ewes ____ Lambs ____

27. Please select the extent to which you agree or disagree with each of the following statements:

	Strongly agree	Agree	Neither agree nor disagree	Disagree	Strongly disagree	Don't know
Blackloss is an inevitable part of hill sheep systems	☐	☐	☐	☐	☐	☐
Understanding the causes of blackloss would help me to reduce lamb losses	☐	☐	☐	☐	☐	☐
Reducing blackloss on my farm/croft is important to me	☐	☐	☐	☐	☐	☐
The threat to lambs from predators on my farm/croft is low	☐	☐	☐	☐	☐	☐

28. Please fill in any other relevant concerns/comments you have:

<u>Section Five: Your contact details (Please complete if you wish to receive feedback/updates on this study)</u>

29. Name: *(Please print)*

30. Contact details:

31. Address and post code:

Thank you for taking the time to complete this survey.

Please return completed forms to:

Fiona Mc Auliffe,
SRUC, The Roslin Institute,
Easter Bush, Midlothian, EH25 9RG

Or email a scanned copy to:

Fiona.McAuliffe@sruc.ac.uk
