# Supplementary material for: Investigating hill sheep farmers and crofters’ experiences of blackloss in the Highlands and Islands of Scotland
Source: PLoS One. 2024 Mar 27;19(3):e0298255. doi: 10.1371/journal.pone.0298255 (PMC10971779; doi:10.1371/journal.pone.0298255)
Supplement: Attachment — Submitted filename: Comments.docx [file pone.0298255.s002.docx]

Dear Editor, Dr. Julio Souza

Thank you for your request dated 25 November 2023 concerning the evaluation of manuscript number PONE-D-23-31486, entitled (**A survey of hill sheep farmer and crofter’s experiences of blackloss in the Highlands and Islands of Scotland**). Bellow my comments on this manuscript:

**General comment**: Well written article, has viable information about topic of this manuscript.

**Title** of this manuscript is excellent. **The abstract** is covered this manuscript completely. It contains very long sentences. I suggest that it be rephrased in short sentences.

**Introduction:**  readable, comprehensive, and covering the subject quite right. It is consist of an important references.

**Materials and methods**: perfect.

**Results:** good and clear. I suggest that the authors indicate their findings in detail, especially the results of tables 1 and 4.

**Discussion:** excellent and clear.

**Conclusion**: Authors wrote the conclusion of their study in abbreviated. I suggest that they rewrite the conclusion in detail, based on the results of their study.

**References:** excellent, clear and complete and important references.

- Some references are old such as: 1963, 1964, 1967, 1971, 1986, 1987, 1990. I suggest replace it with modern references.

**Tables:** excellent and clear**.**

**Figures:** excellent and clear**.**
