# Supplementary material for: Investigating hill sheep farmers and crofters’ experiences of blackloss in the Highlands and Islands of Scotland
Source: PLoS One. 2024 Mar 27;19(3):e0298255. doi: 10.1371/journal.pone.0298255 (PMC10971779; doi:10.1371/journal.pone.0298255)
Supplement: Attachment — Submitted filename: Response to Reviewers.docx [file pone.0298255.s003.docx]

Dear Editor,

I am writing in regards to the manuscript titled “A survey of hill sheep farmer and crofter’s experiences of blackloss in the Highlands and Islands of Scotland”, which has recently undergone review.

The purpose of this letter is to respond to reviewer’s and editor’s comments (see below in blue). On behalf of my co-authors, I wish to thank the reviewers for their helpful comments which we have addressed below and within the manuscript.

Kind regards,

Fiona McAuliffe

1. Please ensure that your manuscript meets PLOS ONE's style requirements, including those for file naming.

Figure files and supporting information now saved and uploaded in appropriate format. Revised manuscript follows style requirements.

2. We note that the grant information you provided in the ‘Funding Information’ and ‘Financial Disclosure’ sections do not match.

When you resubmit, please ensure that you provide the correct grant numbers for the awards you received for your study in the ‘Funding Information’ section.

See amended funding statement below.

3. Thank you for stating the following financial disclosure: 
"This study was undertaken as part of a PhD studentship, funded by Scotland’s Rural College (SRUC) and NatureScot and registered at the University of Edinburgh’s Royal (Dick) School of Veterinary Studies. It involves close collaboration between SRUC, The Royal (Dick) School of Veterinary Studies, NatureScot and SRUC Veterinary Services." 
Please state what role the funders took in the study.  If the funders had no role, please state: ""The funders had no role in study design, data collection and analysis, decision to publish, or preparation of the manuscript."" 
If this statement is not correct you must amend it as needed.

Please include this amended Role of Funder statement in your cover letter; we will change the online submission form on your behalf.

Role of Funder: This study was undertaken as part of a PhD studentship, funded by Scotland’s Rural College (SRUC) and NatureScot. The PhD is registered at the University of Edinburgh’s Royal (Dick) School of Veterinary Studies. It involves close collaboration between SRUC, The Royal (Dick) School of Veterinary Studies, NatureScot and SRUC Veterinary Services. The funders had no role in study design, data collection and analysis, decision to publish, or preparation of the manuscript.

4. Thank you for stating the following in the Acknowledgments Section of your manuscript: 
"The authors wish to thank all those who published or disseminated information about our 
questionnaire. We also wish to thank the hill sheep farmers and crofters who responded to our 
questionnaire. The authors wish to acknowledge Mark Bronsvoort, Margo Chase-Topping and Claire 
Morgan-Davies for their valuable guidance during the typology analysis. This study is part of a PhD 
funded by SRUC and NatureScot and registered at the University of Edinburgh’s Royal (Dick) School of Veterinary Studies. It involves close collaboration between SRUC, The Royal (Dick) School of Veterinary Studies, NatureScot and SRUC Veterinary Services."

We note that you have provided funding information that is not currently declared in your Funding Statement. However, funding information should not appear in the Acknowledgments section or other areas of your manuscript. We will only publish funding information present in the Funding Statement section of the online submission form.
Please remove any funding-related text from the manuscript and let us know how you would like to update your Funding Statement. Currently, your Funding Statement reads as follows: 
"This study was undertaken as part of a PhD studentship, funded by Scotland’s Rural College (SRUC) and NatureScot and registered at the University of Edinburgh’s Royal (Dick) School of Veterinary Studies. It involves close collaboration between SRUC, The Royal (Dick) School of Veterinary Studies, NatureScot and SRUC Veterinary Services."

Please include your amended statements within your cover letter; we will change the online submission form on your behalf.

Funding Statement: This study was undertaken as part of a PhD studentship, funded by Scotland’s Rural College (SRUC) and NatureScot [SRUC Studentship 1033123: *Investigation of causes of lamb loss on Highland farms and crofts*]. The PhD is registered at the University of Edinburgh’s Royal (Dick) School of Veterinary Studies. It involves close collaboration between SRUC, The Royal (Dick) School of Veterinary Studies, NatureScot and SRUC Veterinary Services. The funders had no role in study design, data collection and analysis, decision to publish, or preparation of the manuscript.

Amended Acknowledgments: The authors wish to thank all those who published or disseminated information about our questionnaire. We also wish to thank the hill sheep farmers and crofters who responded to our questionnaire. The authors wish to acknowledge Mark Bronsvoort, Margo Chase-Topping and Claire Morgan-Davies for their valuable guidance during the typology analysis. This study is part of a PhD which involves close collaboration between Scotland’s Rural College, The Royal (Dick) School of Veterinary Studies, NatureScot and SRUC Veterinary Services.

5. Please include your full ethics statement in the ‘Methods’ section of your manuscript file. In your statement, please include the full name of the IRB or ethics committee who approved or waived your study, as well as whether or not you obtained informed written or verbal consent. If consent was waived for your study, please include this information in your statement as well.

We have added a new ‘ethics statement’ subsection to the Methods to address this.

6. Please include a copy of Table 7 which you refer to in your text on page 13 in PDF submission.

This was a typo and has been amended to Table 6.

7. Please review your reference list to ensure that it is complete and correct. If you have cited papers that have been retracted, please include the rationale for doing so in the manuscript text, or remove these references and replace them with relevant current references. Any changes to the reference list should be mentioned in the rebuttal letter that accompanies your revised manuscript. If you need to cite a retracted article, indicate the article’s retracted status in the References list and also include a citation and full reference for the retraction notice.

The reference list has been reviewed, and updated to contain additional modern references as suggested by Reviewer 1.

**Reviewer 1:**

**General comment**: Well written article, has viable information about topic of this manuscript.

**Title** of this manuscript is excellent.

**The abstract** is covered this manuscript completely. It contains very long sentences. I suggest that it be rephrased in short sentences.

The abstract has been edited to break sentences into shorter sentences to improve clarity.

**Introduction:**  readable, comprehensive, and covering the subject quite right. It is consist of an important references.

**Materials and methods**: perfect.

**Results:** good and clear. I suggest that the authors indicate their findings in detail, especially the results of tables 1 and 4.

Further detail has been added when presenting the results from Tables 1 and 4.

**Discussion:** excellent and clear.

**Conclusion**: Authors wrote the conclusion of their study in abbreviated. I suggest that they rewrite the conclusion in detail, based on the results of their study.

Further detail has been added to the conclusion section to reiterate the key findings and results of the study.

**References:** excellent, clear and complete and important references.

- Some references are old such as: 1963, 1964, 1967, 1971, 1986, 1987, 1990. I suggest replace it with modern references.

Some additional modern references have been added (Fisher and Matthews, 2001). Others are seminal works are should be referenced. See below for explanations on each reference.

**Tables:** excellent and clear**.**

**Figures:** excellent and clear**.**

**Reviewer 2:**

The paper present a well planned and carried out survey with some interesting results that could be relevant for ameliorate the mortality rate of lambs in extensive productive system in Scotland.

I only have found a sentence that have no meaning for me, and I recommend to be reword or explain in a different way and is in Lines 304 to 306: “A single respondent from Group 1 reported lamb counts for marking and weaning, with a loss of 1.3%. This is far below the reported estimated losses of 11-20% for Group 1 on a whole, and the >30% loss reported by this respondent, indicating that losses may be overestimated.” It is not clear for me if the same respondent of Group 1 reported lamb losses of 1.3 % and the same respondent reported more that 30% loss. This those do not make sense to me.

This sentence has been reworded to improve clarity. From the lamb counts the study respondent returned we were able to calculate a loss of 1.3%, however the respondent themselves estimated their losses to be >30%.

References:

5 Kaufman L, Rousseeuw PJ. Partitioning Around Medoids (Program PAM). In: Kaufman L, Rousseeuw PJ, editors. Finding Groups in Data: An Introduction to Cluster Analysis. Hoboken, NJ, USA: John Wiley & Sons, Inc.; 1990. pp. 68–125. doi:10.1002/9780470316801

The is the seminal first paper to describe partitioning around medoids, which is the clustering method used in this study, and should therefore be reference.

6 MacQueen J. Classification and analysis of multivariate observations. Fifth Berkeley Symposium on Mathematical Statistics and Probability. Los Angeles LA USA: University of California.; 1967. pp. 281–297.

This is the seminal paper describing k-means clustering, an alternative clustering method, and should therefore be included in the references.

14 Gower JC. A General Coefficient of Similarity and Some of Its Properties. Int Biometric Soc. 1971;27: 857–871. doi:10.2307/2528823

This is the seminal work which described Gower’s distance matrix, which is what was used in the current analysis, and so this paper muct be referenced.

20 Hunter RF, Milner C. The behaviour of individual, related and groups of South Country Cheviot hill sheep. Anim Behav. 1963;11: 507–513. doi:10.1016/0003-3472(63)90270-7

21 Lawrence AB. Mother-daughter and peer relationships of Scottish hill sheep. Anim Behav. 1990;39: 481–486. doi:10.1016/S0003-3472(05)80412-9

The 1963 and 1990 papers descibe sheep home range behaviour in a Scottish context, relvant to the current study and will therefore be retained. Although they are older references they are accompanied by more modern references from 2001 and 2016.

22 Lockie JD. The breeding density of the golden eagle and fox in relation to food supply in Wester Ross, Scotland. Scottish Nat. 1964;71: 67-77.

23 Leitch AF. Report on eagle predation on lambs in the Glenelg area in 1986. 1986.

24 Matchett MR, O’Gara BW. Methods of controlling golden eagle depredation on domestic sheep in southwestern Montana. J Raptor Res. 1987;21: 85–94.

We feel that these references, particularly Lockie (1964) and Leitch (1986) were very important as some of the earliest investigations into eagle depredation of lambs within a Scottish context. Matchett (1987) provides examples of eagle predation of lambs from Montana, USA, however as the sheep husbandry practices are similar to Scottish hill farming it provides a good comparison, and is worthy of inclusion. Several other modern references (2004, 2010, 2013, 2015, and 2021) with relation to eagle predation are also provided.
